# Comparing amikacin and kanamycin-induced hearing loss in multidrug-resistant tuberculosis treatment under programmatic conditions in a Namibian retrospective cohort

**DOI:** 10.1186/s40360-015-0036-7

**Published:** 2015-12-10

**Authors:** Evans L. Sagwa, Nunurai Ruswa, Farai Mavhunga, Timothy Rennie, Hubert G. M. Leufkens, Aukje K. Mantel-Teeuwisse

**Affiliations:** Utrecht Institute for Pharmaceutical Sciences, Utrecht University, Utrecht, The Netherlands; National Tuberculosis and Leprosy Program, Windhoek, Namibia; University of Namibia School of Pharmacy, Windhoek, Namibia

**Keywords:** MDR-TB, Aminoglycosides, Adverse events, Pharmacovigilance, Audiometry

## Abstract

**Background:**

Amikacin and kanamycin are mainly used for treating multidrug-resistant tuberculosis (MDR-TB), especially in developing countries where the burden of MDR-TB is highest. Their protracted use in MDR-TB treatment is known to cause dose-dependent irreversible hearing loss, requiring hearing aids, cochlear implants or rehabilitation. Therapeutic drug monitoring and regular audiological assessments may help to prevent or detect the onset of hearing loss, but these services are not always available or affordable in many developing countries. We aimed to compare the cumulative incidence of hearing loss among patients treated for MDR-TB with amikacin or kanamycin-based regimens, and to identify the most-at-risk patients, based on the real-life clinical practice experiences in Namibia.

**Methods:**

We conducted a retrospective cohort study of patients treated with amikacin or kanamycin-based regimens in four public sector MDR-TB treatment sites in Namibia between June 2004 and March 2014. Patients were audiologically assessed as part of clinical care. The study outcome was the occurrence of any hearing loss. Data were manually extracted from patients’ treatment records. We compared proportions using the Chi-square test; applied stratified analysis and logistic regression to study the risk of hearing loss and to identify the most-at-risk patients through effect-modification analysis. A *P*-value < 0.05 was statistically significant.

**Results:**

All 353 patients had normal baseline hearing, 46 % were HIV co-infected. Cumulative incidence of any hearing loss was 58 %, which was mostly bilateral (83 %), and mild (32 %), moderate (23 %), moderate-severe (16 %), severe (10 %), or profound (15 %). Patients using amikacin had a greater risk of developing the more severe forms of hearing loss than those using kanamycin (adjusted odds ratio (OR) = 4.0, 95 % CI: 1.5–10.8). Patients co-infected with HIV (OR = 3.4, 95 % CI: 1.1–10.6), males (OR = 4.5, 95 %1.5–13.4) and those with lower baseline body weight (40–59 kg, OR = 2.8, 95 % CI: 1.1–6.8), were most-at-risk of developing hearing loss.

**Conclusion:**

Amikacin use in the long-term MDR-TB treatment led to a higher risk of occurrence of the more severe forms of hearing loss compared to kanamycin use. Males, patients with low baseline body weight and those co-infected with HIV were most-at-risk. MDR-TB treatment programmes should consider replacing amikacin with kanamycin and strengthen the routine renal, serum therapeutic drug levels and audiometric monitoring in the most-at-risk patients treated with aminoglycosides.

**Electronic supplementary material:**

The online version of this article (doi:10.1186/s40360-015-0036-7) contains supplementary material, which is available to authorized users.

## Background

Amikacin and kanamycin belong to a group of antibiotics called aminoglycosides, which are used in the treatment of Gram-negative bacterial and mycobacterial infections. These aminoglycosides, in combination with fluoroquinolones, form the backbone for the treatment of multidrug-resistant tuberculosis (MDR-TB), as recommended by the World Health Organization (WHO) [1–3]. A major safety concern of the aminoglycosides is their ability to induce ototoxicity, especially during their long-term use in MDR-TB treatment [4–6]. Depending on the part of the inner ear that is affected as well as the selectivity of the aminoglycoside, the ototoxicity could be auditory or vestibular [7]. The current study focusses on the auditory toxicity (hearing loss or deafness) caused by amikacin and kanamycin. Aminoglycoside-induced hearing loss is permanent, although in some cases; it may be alleviated by the use of hearing aids, cochlear implants or speech rehabilitation, which unfortunately, are costly interventions. By experiencing hearing loss, patients end-up suffering from a distressful yet preventable drug-related disability that may negatively impact on their quality of life and limit their capability to work, for example, in occupations where good hearing ability is a requirement. In children, speech development may be severely compromised [8].

Aminoglycosides have a narrow therapeutic index; hence require careful monitoring of serum levels, particularly during their prolonged use in MDR-TB treatment, to prevent the occurrence of dose-dependent ototoxicity [9, 10]. In addition, regular audiologic assessments may help in the early detection of hearing impairment, before the damage becomes extensive and irreversible [11–13]. Some patients are genetically predisposed to suffering from aminoglycoside-induced hearing loss and genetic typing may be useful in identifying such patients [14–17]. Yet many patients in developing countries do not have access to such interventions or cannot afford them, due to weak public sector health systems and high levels of poverty [18].

Namibia is a developing country situated in the south-western part of Africa. It is classified by the World Bank as an upper middle income country [19]. At the time of this study, there were 13 regional centers for treating patients diagnosed with MDR-TB. One of the centers - the Walvis Bay MDR-TB treatment site - began assessing patients for aminoglycoside-induced hearing loss in 2004. In 2008, Namibia changed the preferred aminoglycoside for MDR-TB treatment from amikacin to kanamycin - which was cheaper and more readily available—and introduced capreomycin as an option for patients prone to hearing loss. Later in the same year, other MDR-treatment sites commenced the systematic audiometric monitoring of patients on MDR-TB treatment for the early detection and management of aminoglycoside-induced hearing loss. The change from amikacin to kanamycin; and the introduction of systematic audiometry provided us with the opportunity of comparing the incidence of hearing loss in patients treated with amikacin and kanamycin-based MDR-TB regimens respectively, in real-life programmatic conditions.

Even though amikacin and kanamycin have been in clinical use for over 50 years, surprisingly to-date, the evidence on their comparative risk of inducing hearing loss is scarce. Moreover, studies often have not been well done, especially in terms of measuring the hearing loss and patients with human immunodeficiency virus (HIV) co-infection have been underrepresented [6]. In sub-Sahara Africa, where the HIV and TB burden are still high [20, 21], HIV co-infection becomes a key consideration in the successful treatment of MDR-TB [22]. Tuberculosis patients co-infected with HIV are an important subgroup because of the potential effect of HIV and antiretroviral treatment on hearing function [23–27].

The aim of this study was to compare the cumulative incidence of hearing loss among patients treated for MDR-TB using amikacin or kanamycin-based regimens, and to identify those that were most-at-risk. The high prevalence of HIV co-infection among patients diagnosed with MDR-TB in Namibia during the period of the study also enabled us to examine the influence of HIV infection on the risk of aminoglycoside-induced hearing loss.

## Methods

### Study design and setting

We conducted a retrospective cohort study of MDR-TB patients treated with amikacin or kanamycin-based regimens between June 2004 and March 2014 at four public sector MDR-TB treatment sites in Namibia. Our study included the four high burden sites that collectively treated over 70 % of the MDR-TB cases in Namibia, during the study period. These were the Katutura, Oshakati, Rundu, and Walvis Bay MDR-TB treatment facilities.

### Study population and sample description

The study population comprised of patients receiving treatment for MDR-TB at public sector facilities in Namibia. Our study sample included all patients who were clinically assessed and audiologically tested for hearing function at baseline and at least once, after commencing their MDR-TB treatment. Patients presenting with symptoms of hearing loss prior to the start of MDR-TB treatment were excluded from our cohort. Upon suspicion or after being diagnosed with MDR-TB infection, patients were initiated on six-to-eight months of intensive phase treatment with a regimen that contained either amikacin or kanamycin, until two sputum smears and two successive cultures turned negative for *Mycobacterium tuberculosis*. Thereafter, treatment was changed to the continuous phase for 12–18 months that was administered on an outpatient basis. The average daily patient dose of amikacin or kanamycin was 15 mg per kilogram body weight, although dosing could be adjusted depending on the patient age group, weight band and renal function [2]. Patients were tested for HIV infection and, if infected, were enrolled on highly active antiretroviral treatment according to the Namibian HIV treatment guidelines that were current at that time [28].

### Study outcome

The occurrence of hearing loss after initiation of MDR-TB treatment was the main outcome of this study and was determined by an audiologist using pure tone audiometry as part of the usual care of patients treated for MDR-TB infection at the sites. Audiometry was performed at baseline, during the intensive phase of MDR-TB treatment and also in the continuation phase. No audiometry was done after completion of the MDR-TB treatment for patients who did not develop hearing loss. Hearing ability was tested by establishing the lowest intensity of sound in decibels (dB) that the person could hear at successive frequencies from 250 Hertz to 8,000 Hertz. Based on the audiogram chart provided by the Namibian Ministry of Health and Social Services (Additional file 1), the level of hearing was classified as normal (0–20 dB), mild (21–40 dB), moderate (41–60 dB), moderate-to-severe (61–80 dB), severe (81–100 dB), or profound (101–120 dB). Although the thresholds are not exactly the same, this classification of the severity of hearing loss is similar to the one provided by the American Speech-Language-Hearing Association (ASHA) [29].

The potential confounders or effect modifiers of the aminoglycoside exposure and hearing loss relationship were patients’ baseline age and weight, sex, renal function, HIV status, year of treatment initiation and the treatment site. Since capreomycin was reserved for use in patients considered at risk of developing hearing loss at the start, or at any time in the course of the intensive phase of the MDR-TB treatment, its use was not included in our study, to guard against confounding by indication of this drug.

### Data abstraction and processing

Data were abstracted from clinical records using a structured form, single-entered into Epi Info™ Version 7.1.4 software (July 2014; Centers for Disease Control and Prevention, Atlanta, GA, USA) and the accuracy of entry verified against the original paper forms. All patient names and other identifiers were omitted from the final dataset to protect their privacy and to ensure their confidentiality. The anonymized and de-identified patient records were analyzed and reported in an aggregate manner, except for one patient whose serial audiograms have been anonymously published.

### Data analysis

Data were summarized using descriptive statistics. We compared continuous variables using the Student’s *t*-test and categorical variables using the Chi-square test or the Fisher exact test. We performed univariable unconditional logistic regression analysis to assess the relationship between aminoglycoside use (amikacin or kanamycin) and the occurrence of any hearing loss. We repeated the same analysis for the less severe forms of hearing loss (mild or moderate); and the more severe forms of hearing loss (moderate-to-severe, severe, or profound). We performed stratified analyses to assess effect modification by patients’ age group, sex, baseline body weight band and HIV status. The Breslow-Day test of homogeneity was used to determine if the strata-specific odds ratios were similar. Multivariable logistic regression was conducted to adjust for potential confounders for variables whose *P*-value for the association with hearing loss was < 0.2. We used Epi Info™ Version 7.1.4 software (July 2014; Centers for Disease Control and Prevention, Atlanta, GA, USA), for the analysis.

### Ethics statement

The study was approved by the institutional review board (IRB) of the Utrecht University (Reference: UP1307) and the research and ethics committee of the Namibian Ministry of Health and Social Services, (MoHSS), (Reference: 17/3/3). A waiver of the requirement for informed consent from the patients was requested from the IRB and the MoHSS, because the study involved the review and analysis of clinical data that are routinely collected as part of the usual medical care of patients being treated for MDR-TB in Namibia. All patient names and other identifiers were omitted from the final dataset to protect their privacy and to ensure their confidentiality.

## Results

There were 353 patients whose records were retrieved, all of whom had documented normal hearing at the start of their tuberculosis treatment. Fifty one (14 %) of the patients were treated with amikacin-based regimens and 302 (86 %) with kanamycin-based regimens. There were 164 patients (46 %) who were HIV co-infected, of whom 132 (80 %) were on highly active antiretroviral treatment. These patient characteristics were comparable between the amikacin and kanamycin-exposed groups (Table 1).

**Table 1:** Baseline characteristics of the patients, by aminoglycoside exposure

	All cases (*N* = 353)		
	Amikacin (*n* = 51)	Kanamycin (*n* = 302)	*P*- value
Age (years): mean ± SD	35.69 ± 9.56	36.47 ± 11.57	*P* = 0.85
Body weight (kgs): mean ± SD	49.58 ± 8.83	50.76 ± 12.0	*P* = 0.77
Sex:			
Male, n (%)	32 (63 %)	166 (55 %)	*P* = 0.47
Female, n (%)	19 (37 %	136 (45 %)	
HIV co-infection: n (%)	25 (49 %)	139 (46 %)	*P* = 0.53
DR-TB site:			
Katutura	0	46 (15 %)	
Oshakati	1 (2 %)	65 (22 %)	*P* < 0.001
Rundu	0	67 (22 %)	
Walvis Bay	50 (98 %)	124 (41 %)	
Reporting period:			
2004–2009, n (%)	45 (88 %)	48 (16 %)	*P* < 0.001
2010–2011, n (%)	4 (8 %)	61 (20 %)	
2012, n (%)	1 (2 %)	112 (37 %)	
2013–2014, n (%)	0	80 (27 %)	
Missing, n (%)	1 (2 %)	1 (0.3 %)	

*SD* standard deviation, *IQR* interquartile range, *HIV* Human immunodeficiency virus

**Fig. 1:**
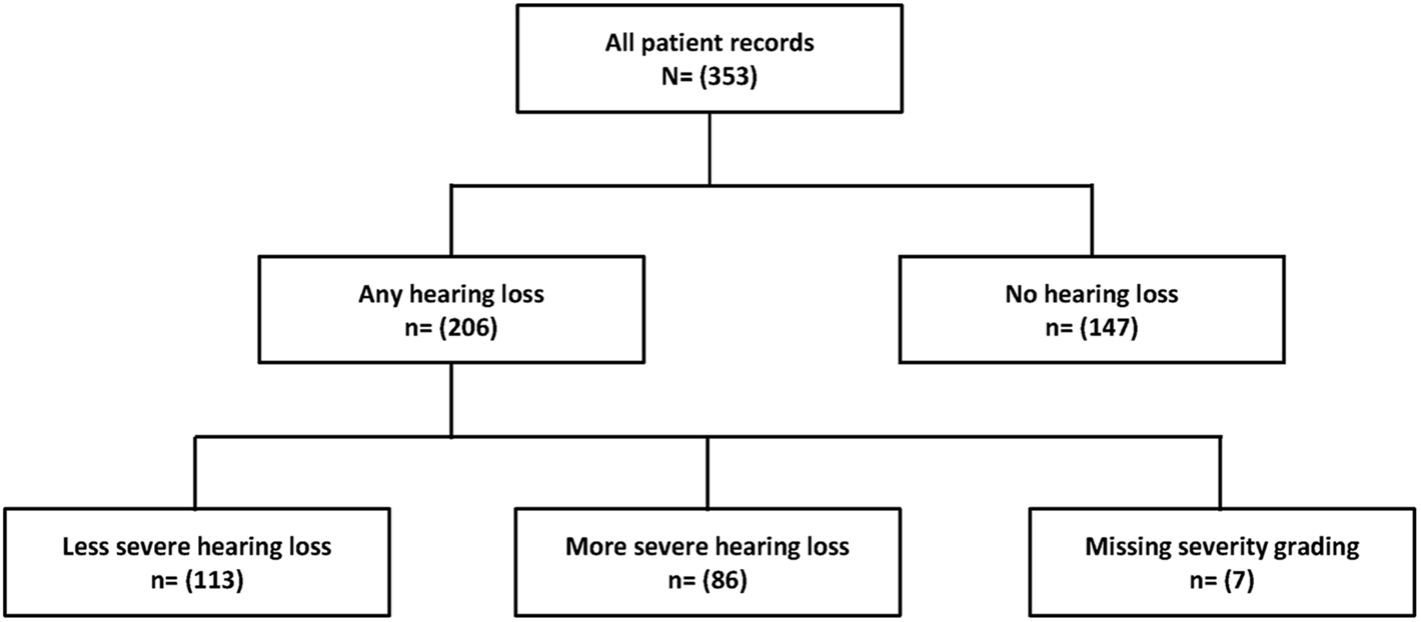
Study flow diagram depicting the presence or absence of hearing loss, by severity, in patients treated for MDR-TB

Subsequently, during the course of their MDR-TB treatment, 206 of the patients (58 %) developed hearing loss of any severity grading (Fig. 1 and Table 2). The hearing loss was mild in 32 % of the patients, moderate (23 %), moderate-severe (16 %), severe (10 %), or profound (15 %) as shown in Table 2. Two-thirds (66 %) of the patients with audiometrically confirmed hearing damage needed to be fitted with an hearing aid or to undergo speech rehabilitation.

**Table 2:** The cumulative incidences of severity of hearing loss by type of aminoglycoside in the MDR-TB drug regimen

Severity of hearing loss	Amikacin group (*n* = 51)	Cumulative incidence	Kanamycin group (*n* = 302)	Cumulative incidence	Total cases (*N* = 353)	Cumulative incidence
Mild	5	10 %	60	20 %	65	18 %
Moderate	7	14 %	41	14 %	48	14 %
Moderate-Severe	6	12 %	27	9 %	33	9 %
Severe	5	10 %	16	5 %	21	6 %
Profound	11	22 %	21	7 %	32	9 %
Missing severity grading	4	8 %	3	1 %	7	2 %
Total cases	38	75 %	168	56 %	206	58 %

**Fig. 2:**
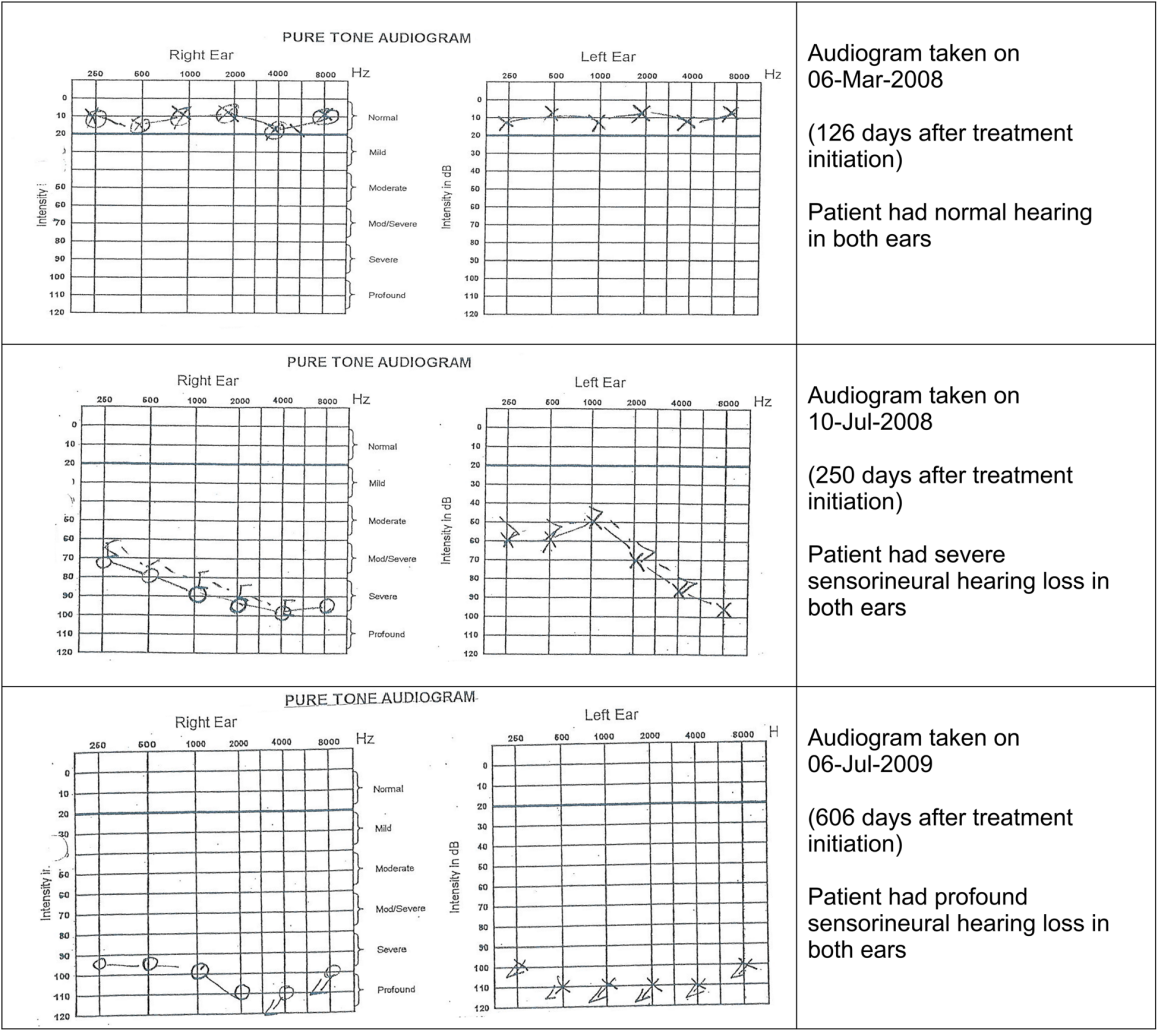
Serial audiograms for patient X, who developed profound hearing loss during MDR-TB treatment

The hearing loss was sensorineural and predominantly bilateral (83 %), always beginning with high frequency loss (4–8 kHz), and then progressing to involve the lower frequencies (0.25–3 kHz) that are used for speech and conversation as shown in one of the patient’s audiogram in Fig. 2. Patient X was a 36 years old male, weighing 53 kg at the start of MDR-TB treatment. His treatment regimen for the intensive phase contained amikacin, ethambutol, ethionamide and pyrazinamide. The patient began experiencing loss of hearing after about four months of treatment with this regimen. Amikacin was stopped, but the patient continued experiencing the hearing loss after the cessation of amikacin. He later developed profound hearing loss long after treatment with amikacin was stopped.

The cumulative incidence of any hearing loss was greater among patients treated with amikacin-based regimens than in those treated with kanamycin-based regimens (75 % versus 56 %, *p* = 0.01), (Table 3), and the difference was largest for profound hearing (amikacin, 22 % versus kanamycin, 7 %, *p* = 0.01). Patients treated with amikacin had more than twice the odds of kanamycin of developing any hearing loss (crude odds ratio (OR) = 2.3; 95 % CI 1.2–4.6), although the confidence interval for the odds ratio for the association became wider after adjusting for confounders (adjusted OR = 2.3; 95 % CI 1.0–5.4) as shown in Table 4. When the severity of the hearing loss was taken into consideration, patients treated with amikacin had a significantly greater risk of experiencing the more severe forms of hearing loss (adjusted OR = 4.0, 95 % CI: 1.5–10.8), than of developing the less severe forms (adjusted OR = 1.6, 95 % CI: 0.6–4.5).

**Table 3:** Amikacin versus kanamycin use in MDR-TB treatment and the presence or absence of hearing loss

Aminoglycoside exposure	Any hearing loss	No hearing loss	Total
Kanamycin	168 (56 %)	134 (44 %)	302
Amikacin	38 (75 %)	13 (25 %)	51
Total	206	147	353

**Table 4:** Relative risk of hearing loss of amikacin and kanamycin use in MDR-TB treatment

	Any hearing loss Crude OR (95 % CI)	Any hearing loss ^a^aOR (95 % CI)	Less severe hearing loss ^a^aOR (95 % CI)	More severe hearing loss ^a^aOR (95 % CI)
Kanamycin	Reference	Reference	Reference	Reference
Amikacin	2.3 (1.2–4.6)	2.3 (1.0–5.4)	1.6 (0.6–4.5)	4.0 (1.5–10.8)

Legend: *aOR* adjusted odds ratio, ^a^adjusted for patient age, treatment site and year of treatment initiation; 95%CI =95 % confidence interval. Note that baseline body weight band, sex, human immunodeficiency virus infection status were not adjusted for because they were potential effect-modifiers (see Table 5)

In stratified analyses by patients’ age group, baseline body weight band and by HIV infection status respectively, we noticed differences in the odds ratios of the amikacin/kanamycin and hearing loss relationship in the different strata of these variables. Although effect modification could not be statistically confirmed due to low numbers, patients co-infected with HIV (crude OR = 3.4, 95 % CI: 1.1–10.6), males (crude OR = 4.5, 95 %1.5–13.4) and those weighing 40–59 kg (crude OR = 2.8, 95 % CI: 1.1–6.8), appeared to be at higher risk of developing hearing loss (Table 5).

**Table 5:** Effect-modification of the amikacin or kanamycin exposure and the occurrence of hearing loss in MDR-TB treatment

Variable	Strata	Stratum-specific crude OR (95 % Confidence Interval)	P-value (Breslow-Day test of homogeneity across strata)
Age	Age 0–24 years	2.0 (0.4–10.6)	
	Age 25–34 years	2.8 (0.8–9.2)	0.99
	Age 35–44 years	2.6 (0.8–8.2)	
	Age 45+ years	3.0 (0.4–26.4)	
Baseline body weight	Weight 18–39 kgs	NA	
	Weight 40–59 kgs	2.8 (1.1–6.8)	0.06
	Weight 60+ kgs	1.1 (0.4–3.0)	
Sex	Female	1.1 (0.4–3.0)	0.06
	Male	4.5 (1.5–13.4)	
HIV status	HIV negative	1.7 (0.7–4.1)	
	HIV positive	3.4 (1.1–10.6)	0.33

Legend: *NA* not possible to estimate due to some cells having zero values, *kgs* kilograms, *HIV* human immunodeficiency virus infection status

## Discussion

Adverse drug reactions are an important consideration for patients treated for MDR-TB infection where the prolonged treatment with amikacin or kanamycin is likely to result in the development of permanent hearing loss [16]. We report a high incidence of aminoglycoside-induced hearing loss, which was more frequent in patients treated with amikacin-based regimens than in those containing kanamycin. The high cumulative incidence of hearing loss (75 %) in the amikacin-exposed group in our setting is similar to the 70 % that was reported by Reza Javadi et. al. [30], while the 56 % incidence for kanamycin is similar to the 58 % reported by Sataloff and colleagues [31]. This provides compelling evidence that amikacin is more ototoxic than kanamycin, in real-life clinical practice.

To the best of our knowledge, the current study is the first one to quantify the comparative risk of hearing loss of amikacin versus kanamycin in their real-life use for MDR-TB treatment in a low-resource setting. It builds on previous research from other settings, which suggested that amikacin was associated with a greater risk of hearing loss, but did not quantify the magnitude of that risk [16]. Our finding is corroborated by the works of Duggal and Sarkar as well as by Sturdy and colleagues [16, 18] . In Duggal and Sarkar’s study, seven out of 34 patients (20.6 %) treated with amikacin for MDR-TB experienced sensorineural hearing loss involving the higher frequencies while a lesser proportion of four out of 26 patients (15.4 %) treated with kanamycin experienced the same type of hearing loss [18]. Similarly, Sturdy et.al. monitored the occurrence of hearing loss in 50 MDR-TB patients, 29 of whom were treated with amikacin, 11 with capreomycin and 10 with streptomycin, and found that the use of amikacin (*P* = 0.02) and decreased renal function (*P* = 0.01) were significantly associated with the development of hearing loss [16]. Although both studies involved small numbers of patients, their findings have been crucial in elucidating on the relative ototoxicity of the aminoglycosides used in MDR-TB treatment. Considering our current findings and those of previous research, we encourage clinicians and managers of the TB control programs that are still using amikacin as the preferred aminoglycoside for treating MDR-TB infection, to consider changing to kanamycin. Switching to kanamycin and implementing other preventive measures, will help to reduce the occurrence of aminoglycoside-induced hearing loss among patients treated for MDR-TB.

The hearing loss seen in our study was sensorineural, mostly bilateral and began by affecting higher frequencies, then progressing to lower conversational-level frequencies as the severity of deafness increased. This finding is consistent with the pathophysiology of aminoglycoside-induced hearing loss [4, 9]. After parenteral administration, aminoglycosides enter the inner ear fluids of the organ of Corti and the sensory hair cells where they are thought to react with heavy metal ions to form highly reactive free radicals that damage the stereocilia of the sensory hair cells [32, 33]. There is emerging evidence that the use of antioxidants like salicylates, ion chelating agents or calcium-binding proteins may prevent aminoglycoside-induced hearing loss [15, 34–37]. As illustrated in the case of patient X in this paper, a patient’s hearing ability could continue deteriorating even after withdrawing the aminoglycoside due to the long half-life or the sequestration of aminoglycosides in the endolymph of the cochlea canals, which continues to cause the loss of sensory hair cells long after stopping the administration of the drug [7, 38].

We, therefore, advocate for MDR-TB treatment programs to implement routine serial audiometry in patients treated with aminoglycosides even in resource constrained settings, so that patients showing early signs of hearing loss can be identified long before the damage is too late to be reversed. When the drugs for preventing aminoglycoside-induced hearing loss become licensed for clinical use, they should be readily made available to patients, as an additional means of protecting patients from developing aminoglycoside-induced hearing loss.

The risk of aminoglycoside-induced hearing loss was greatest in patients with lower baseline body weight (40–59 kg). This could be due to a drug dosing problem whereby clinicians may fail to titrate accurately the aminoglycoside doses according to individual patient body weight. Alternatively, these could be patients who were much sicker of tuberculosis disease than the heavier weighing patients. Since we are unable to ascertain the reason for this observation due to lack of data on serum drug concentrations, we recommend further studies on the long-term pharmacokinetics and pharmacodynamics of aminoglycosides in the context of MDR-TB treatment, taking into consideration patients’ renal function, anthropometric and genetic characteristics.

Patients co-infected with HIV were more at risk of amikacin-induced hearing loss than the HIV uninfected ones. There is emerging epidemiologic and clinical evidence about the association between HIV infection and hearing loss [27, 38, 39]. However, whether antiretroviral medicines also induce hearing loss is a question that is still unanswered because of the mixed findings of previous studies [25, 26, 40]. Besides, the current study doesn’t shed light on this question because of a lack of adequate data on the specific antiretroviral (ARV) drug regimens used by the patients and insufficient patient numbers, by ARV regimen. There is need for continued research in this area to better understand the effect of antiretroviral medicines on hearing ability.

Amikacin, kanamycin and other aminoglycosides are practically not metabolized by the human body and are excreted unchanged almost exclusively by glomerular filtration, hence they require the careful monitoring of their plasma levels during therapy [41, 42]. Unfortunately, therapeutic drug monitoring (TDM) of the aminoglycoside plasma levels was not performed during the treatment of patients for MDR-TB infection in Namibia. This service was not available in the public sector health system in Namibia and is not available in many developing countries [43], perhaps explaining the relatively high incidence of ototoxicity reported among patients on MDR-TB treatment in these countries. We recommend that TB treatment programs in developing countries should consider introducing routine therapeutic drug monitoring for patients treated with aminoglycosides or capreomycin, given the higher cost of correcting permanent hearing loss for the patient and the society. A comparative cost-effectiveness analysis of conducting TDM versus not doing TDM can further inform such a strategy.

Renal clearance may strongly affect the toxicity of aminoglycosides [41, 42, 44]. The lack of data on renal clearance for the patients included in our analysis is an important limitation of the current study. Although we retrieved the serum creatinine levels of 114 of the 353 patients from the laboratory database, the data was of no benefit to this analysis because it represented creatinine values that were measured at time points several months after the initiation of the MDR-TB treatment and baseline data were essentially missing. This, however, does not mean that clinicians in Namibia do not assess patients on MDR-TB treatment for renal function. They do so, but because of some practical challenges in the collection of data for this study, we could not retrieve all the data on serum creatinine levels for the patients in our analysis. Nonetheless, we encourage clinicians in our setting to systematically assess all patients on MDR-TB treatment, or those at the greatest risk, for renal function at baseline and over the course of the treatment, as recommended by the Namibian TB treatment guidelines [2].

Our study was an epidemiologic one, reflecting the real-life usage of amikacin and kanamycin in routine clinical practice. Using this study design, we identified patients that were most-at-risk of developing aminoglycoside-induced hearing loss. Importantly, hearing function was assessed using audiograms, which were part of the routine clinical follow-up of patients treated for MDR-TB. However, the data on the time-to-onset of hearing loss was unreliable because of the “batching” of patients for audiometry, due to the shortage of audiologists and audiology assistants.

Due to practical limitations about the documentation of audiometry in patients treated for MDR-TB at the sites prior to 2007, we could only retrieve information on 51 patients that were treated with amikacin for the period covered by this research. On the other hand, for kanamycin, we retrieved 302 patients, causing an imbalance in the numbers of patients exposed to amikacin and kanamycin, respectively. To assess for possible bias, the 51 patients on amikacin were checked for the risk of hearing loss against 51 randomly selected patients on kanamycin and the results were similar to those of the 353 patient sample.

There were several other limitations of this study. For example, there were too few patients in some sub-groups which limited the power of the study for multiple sub-group analyses. Besides, we were unable to collect data on other potential risk factors like the usage of antiretroviral medicines in HIV infected patients, genetic markers of ototoxicity and other unmeasured confounders including the use of other medicines known to be ototoxic.

## Conclusion

The long-term use of amikacin in MDR-TB treatment led to a higher risk of the more severe forms of hearing loss compared to the use of kanamycin for the same indication. Males, patients with low baseline body weight and those co-infected with HIV were most-at-risk. We recommend that managers of MDR-TB treatment programmes should consider using kanamycin instead of amikacin for the treatment of MDR-TB; and invest more resources in building the capacity and skills of health care personnel for routine renal, serum therapeutic drug levels and audiometric monitoring of the most-at-risk patients treated with aminoglycosides. More research needs to be done to better understand the combined risk of hearing loss in patients concomitantly treated for MDR-TB and HIV infections. A better designed and more powered study is needed to confirm the comparative ototoxicity risk of amikacin and kanamycin; and associated risk factors.

## Additional file

Additional file 1:
**Sample audiological assessment form used by the Namibian Ministry of Health and Social Services.** (PDF 488 kb)

## References

[1] World Health Organization (WHO) (2008). Guidelines for the programmatic management of drug-resistant tuberculosis.

[2] Ministry of Health and Social Services (MoHSS) (2012). National Guidelines for the Management of Tuberculosis.

[3] Caminero JA, Sotgiu G, Zumla A, Migliori GB (2010). Best drug treatment for multidrug-resistant and extensively drug-resistant tuberculosis. Lancet Infect Dis.

[4] Brummett RE, Fox KE (1989). Aminoglycoside-induced hearing loss in humans. Antimicrob Agents Chemother.

[5] Cianfrone G, Pentangelo D, Altissimi G, Turchetta R (2011). Pharmacological drugs inducing ototoxicity, vestibular symptoms and tinnitus: a reasoned and updated guide. Eur Rev Med Pharmacol Sci..

[6] Seddon JA, Godfrey-Faussett P, Jacobs K, Ebrahim A, Hesseling AC, Schaaf HS (2012). Hearing loss in patients on treatment for drug-resistant tuberculosis. Eur Respir J..

[7] Selimoglu E (2007). Aminoglycoside-induced ototoxicity. Curr Pharm Des..

[8] Olusanya BO, Neumann J, Saunders JE (2014). The global burden of disabling hearing impairment : a call to action. Bull World Health Organ..

[9] Black RE, Lau WK, Weinstein RJ, Young LS, Hewitt WL (1976). Ototoxicity of Amikacin. Antimicrob Agents Chemother..

[10] World Health Organization (WHO) (1994). Report of an informal consultation on strategies for prevention of hearing impairment from ototoxic drugs.

[11] Jacob LCB, Aguiar FP, Tomiasi AA, Tschoeke SN, De Bitencourt RF (2006). Auditory monitoring in ototoxicity. Braz J Otorhinolaryngol..

[12] Singh Chauhan R, Saxena RK, Varshey S (2011). The role of ultrahigh-frequency audiometry in the early detection of systemic drug-induced hearing loss. Ear Nose Throat J..

[13] Fausti SA, Wilmington DJ, Helt PV, Helt WJ, Konrad-Martin D (2005). Hearing health and care: The need for improved hearing loss prevention and hearing conservation practices. J Rehabil Res Dev..

[14] Bardien S, de Jong G, Schaaf HS, Harris T, Fagan J, Petersen L (2009). Aminoglycoside-induced hearing loss : South Africans at risk. South African Med J..

[15] Perletti G, Vral A, Patrosso MC, Marras E, Ceriani I, Willems P (2008). Prevention and modulation of aminoglycoside ototoxicity (Review). Mol Med Rep..

[16] Sturdy A, Goodman A, José RJ, Loyse A, O’Donoghue M, Kon OM (2011). Multidrug-resistant tuberculosis (MDR-TB) treatment in the UK: a study of injectable use and toxicity in practice. J Antimicrob Chemother..

[17] Verdel BM, van Puijenbroek EP, Souverein PC, Leufkens HGM, Egberts ACG (2008). Drug-related nephrotoxic and ototoxic reactions : a link through a predictive mechanistic commonality. Drug Saf..

[18] Duggal P, Sarkar M (2007). Audiologic monitoring of multi-drug resistant tuberculosis patients on aminoglycoside treatment with long term follow-up. BMC Ear Nose Throat Disord..

[19] World Bank (2015). Namibia | Data [Internet].

[20] UNAIDS. UNAIDS report on the global AIDS epidemic (2013). UNAIDS Global Report, 2013.

[21] World Health Organization (WHO) (2014). Global Tuberculosis Report, 2014.

[22] Venkatesh KK, Swaminathan S, Andrews JR, Mayer KH (2011). Tuberculosis and HIV co-infection: screening and treatment strategies. Drugs..

[23] Monte S, Fenwick JD, Monteiro EF (1997). Irreversible ototoxicity associated with zalcitabine. Int J STD AIDS..

[24] Rey D, L’Héritier A, Lang J (2002). Severe Ototoxicity in a Health Care Worker Who Received Postexposure and Nevirapine after Occupational Exposure to HIV. Clin Infect Dis..

[25] Simdon J, Watters D, Bartlett S, Connick E (2001). Ototoxicity associated with use of nucleoside analog reverse transcriptase inhibitors: a report of 3 possible cases and review of the literature. Clin Infect Dis..

[26] Batista A, Vieira C, Greco DB (2008). Otoneurological manifestations associated with antiretroviral therapy. Rev Soc Bras Med Trop..

[27] Katijah K (2010). Is there a need for ototoxicity monitoring in patients with HIV/AIDS?. African J Pharm Pharmacol..

[28] Ministry of Health and Social Services (MoHSS) (2010). National Guidelines for Antiretroviral Therapy.

[29] ASHA. Degree of Hearing Loss [Internet]. ASHA; [cited 2015 Oct 15]. Available from: http://www.asha.org/public/hearing/Degree-of-Hearing-Loss/. Accessed on 15 October 2015.

[30] Reza M, Abtahi B, Gholami K (2011). The Incidence of Amikacin Ototoxicity in Multidrug-Resistant Tuberculosis Patients. Iran J Pharm Res..

[31] Sataloff J, Wagner S, Menduke H (1964). Kanamycin ototoxicity in healthy men. Arch Otolaryngol..

[32] Huth ME, Ricci AJ, Cheng AG (2011). Mechanisms of aminoglycoside ototoxicity and targets of hair cell protection. Int J Otolaryngol..

[33] Szczepanik W, Kaczmarek P, Jezowska-Bojczuk M (2004). Oxidative activity of copper(II) complexes with aminoglycoside antibiotics as implication to the toxicity of these drugs. Bioinorg Chem Appl..

[34] Sha SH, Schacht J (1997). Prevention of aminoglycoside-induced hearing loss. Keio J Med..

[35] Lopez-Gonzalez MA, Guerrero JM, Torronteras R, Osuna C, Delgado F (2000). Ototoxicity caused by aminoglycosides is ameliorated by melatonin without interfering with the antibiotic capacity of the drugs. J Pineal Res..

[36] Cheng AG, Cunningham LL, Rubel EW (2005). Mechanisms of hair cell death and protection. Curr Opin Otolaryngol Head Neck Surg..

[37] Karasawa T, Wang Q, David LL, Steyger PS (2011). Calreticulin binds to gentamicin and reduces drug-induced ototoxicity. Toxicol Sci..

[38] Harris T, Bardien S, Schaaf HS, Petersen L, Jong G de, Fagan JJ. Aminoglycoside-induced hearing loss in HIV-positive and HIV-negative multidrug-resistant tuberculosis patients. South African Med J. 2012. 363–6. 10.7196/samj.4964 22668907

[39] Harris T, Heinze B (2013). Open Access Guide to Audiology and Hearing Aids for Otolaryngologists.

[40] Schouten JT, Lockhart DW, Rees TS, Collier AC, Marra CM (2006). A prospective study of hearing changes after beginning zidovudine or didanosine in HIV-1 treatment-naïve people. BMC Infect Dis..

[41] Pechere J-C, Dugal R (1979). Clinical Pharmacokinetics of Aminoglycoside Antibiotics. Clin Pharmacokinet..

[42] De Jager P, Van Altena R (2002). Hearing loss and nephrotoxicity in long-term aminoglycoside. Int J Tuberc Lung Dis..

[43] Nwobodo N (2014). Therapeutic drug monitoring in a developing nation: a clinical guide. JRSM Open..

[44] Prayle A, Watson A, Fortnum H, Smyth A (2010). Side effects of aminoglycosides on the kidney, ear and balance in cystic fibrosis. Thorax..

