# Supplementary material for: Comparing amikacin and kanamycin-induced hearing loss in multidrug-resistant tuberculosis treatment under programmatic conditions in a Namibian retrospective cohort
Source: BMC Pharmacol Toxicol. 2015 Dec 10;16:36. doi: 10.1186/s40360-015-0036-7 (PMC4675035; doi:10.1186/s40360-015-0036-7)
Supplement: Additional file 1: — Sample audiological assessment form used by the Namibian Ministry of Health and Social Services. (PDF 488 kb) [file 40360_2015_36_MOESM1_ESM.pdf]

MINISTRY OF HEALTH AND SOCIAL SERVICES

EAR, NOSE AND THROAT DEPARTMENT

AUDIOLOGICAL ASSESSMENT

SURNAME& INITIALS: ....

DATE OF BIRTH: ....

GENDER: .... WARD/DEPT ....

ADDRESS/SCHOOL: ....

TELEPHONE: .... DATE: ....

REFERRED BY: .... TESTER: ....

AUDIOMETER: .... NO. OF TEST: ....

TEST RELIABILITY

Good ....

Fair ....

Poor ....

BACKGROUND INFORMATION: ....

....

PURE TONE AUDIOGRAM

Right Ear

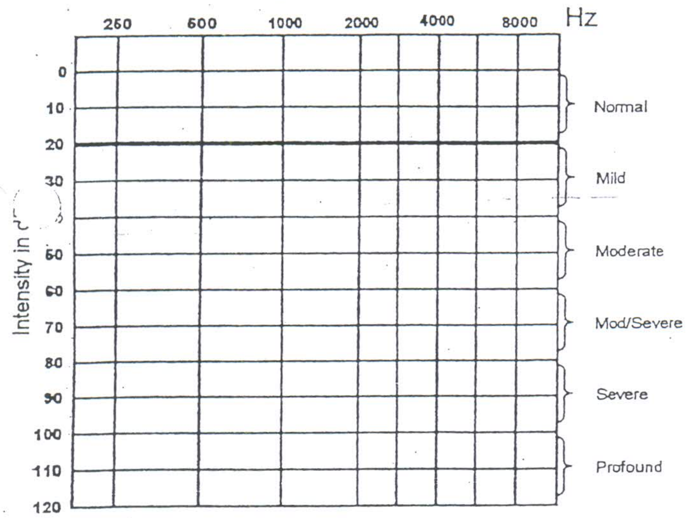

Masking

Air						
Bone						

Left Ear

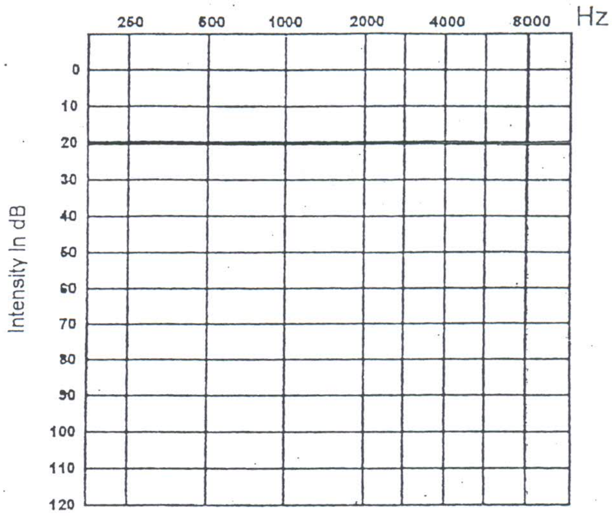

Masking

Air						
Bone						

% Hearing loss (ISO) Right .... % Left .... % Binaural .... % Type of loss: ....

WEBER

250	500	1000	2000	4000

TYMPANOGRAM

	Type	M E P	Compliance	Ear canal vol
Right				
Left				

KEY

	R	L
Air cond.	○	×
Bone cond.	<	>
Masked	◁	▷
No response	↙	↙
Contra reflex	⊠	⊠
Ipsi reflex	□	□

SPEECH AUDIOGRAM Intensity dB

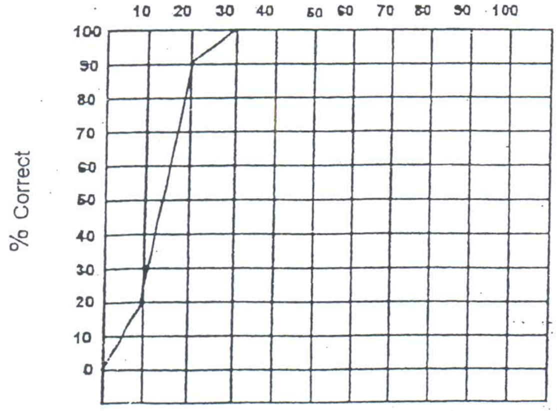

HEARING AID EVALUATION

TYPE		
EAR		
Aid - Lipreading	%	%
Aid + Lipreading	%	%
F.F - Lipreading	%	%
F.F + Lipreading	%	%

REMARKS: ....

....

....

....

....

....

EAR	PTT	SRT	Max. discrimination
LEFT			
RIGHT			
